# Pathological Fractures of the Mandible: Our Department’s 15-Year Experience

**DOI:** 10.3390/diagnostics15101216

**Published:** 2025-05-12

**Authors:** Georgios Chatziantoniou, Dimitris Tatsis, Solon Politis, Antonios Saramantos, Nikolaos Koukolis, Konstantinos Paraskevopoulos

**Affiliations:** 1 Department of Oral & Maxillofacial Surgery, Aristotle University of Thessaloniki, Specialized Cancer Treatment and Reconstruction Center, General Hospital of Thessaloniki “George Papanikolaou”, 57010 Thessaloniki, Greece; saramantosant@gmail.com (A.S.); kostparas@yahoo.gr (K.P.); 2 Oral and Maxillofacial Surgery Clinic, 424 Military Hospital of Thessaloniki, 56429 Thessaloniki, Greece; solonpolitis88@gmail.com; 3 Private Practice, 57010 Thessaloniki, Greece; koukolisn@yahoo.gr

**Keywords:** pathological jaw fracture, MRONJ, ORN, spontaneous fracture, mandible

## Abstract

**Background/Objectives**: Pathological fractures of the mandible are uncommon and often result from underlying conditions such as osteoradionecrosis, malignancies, or medication-related osteonecrosis of the jaw (MRONJ). Their management is challenging due to compromised bone quality and complex patient comorbidities. This study presents a 15-year experience from a tertiary oral and maxillofacial surgery center, highlighting the clinical characteristics, etiologies, treatment approaches, and outcomes of these fractures. **Methods**: A retrospective review was conducted on patients diagnosed with pathological mandibular fractures between 2010 and 2024. Data collected included demographics, fracture etiology and location, diagnostic imaging, treatment modality, complications, and long-term outcomes. **Results**: Fifty patients met the inclusion criteria. The mean age was 66.4 years, with a predominance of male patients (78%). The most common etiology was osteoradionecrosis (48%), followed by primary malignancy (22%) and MRONJ (16%). In 82% of cases, surgical management was required, most frequently involving marginal or segmental mandibular resection (gnathectomy), with or without immediate reconstruction. Conservative treatment was reserved for select cases with high surgical risk. Complications occurred in 54% of patients, including persistent fistulas, pathological communication with the skin or oral cavity, and the need for revision surgery. Long-term follow-up revealed variable survival, with many patients experiencing reduced quality of life due to complex postoperative courses. **Conclusions**: Pathological fractures of the mandible present significant diagnostic and therapeutic challenges, particularly in patients with osteoradionecrosis or malignancies. Early diagnosis and individualized, multidisciplinary treatment planning are essential. This study underscores the need for a standardized classification system and treatment algorithm to guide management and improve outcomes in this complex patient population.

## 1. Introduction

Pathological fractures of the jaws, particularly the mandible, are a unique category of fractures, which are relatively rare, amounting to less than 1% [1]. Most of these data are documented in independent case series and reports. Nonetheless, although infrequent, pathological fractures present a significant clinical challenge because they negatively impact the quality of life for affected patients, as well as needing a possibly complex and patient personalized treatment planning.

Patients presenting with pathological fractures have both functional and aesthetic impairments and are psychologically affected as well. Functionally, these patients experience high levels of chronic pain [2], especially during the mastication process, due to the underlying fracture. In patients with underlying conditions, especially elderly ones, this further deteriorates their well-being [3]. Speech and overall oral hygiene may also be hindered [4,5]. Aesthetically, the facial contour may also be adversely affected, leading to a possible decline in self-esteem and increased anxiety about daily social interactions. Overall, these conditions may exacerbate social withdrawal and mental health issues [6,7,8,9].

Pathological fractures of the mandible occur due to weakened bone integrity from underlying systemic or localized conditions. Pathologic fractures, in general, are predominantly secondary to metastatic disease rather than primary bone malignancies or other conditions [10], with metastatic lesions from lung, breast, thyroid, renal, and prostate cancer being the most common contributors [10,11]. However, mandibular pathologic fractures and their etiology differ in that regard, as the most prominent underlying causal factors involve osteonecrosis of the jaws (osteoradionecrosis and medically related osteonecrosis of the jaws (MRONJ)), tumors, surgical procedures, and infections. Iatrogenic causes are eminent in the majority of the cases, with osteoradionecrosis representing the majority of cases, while exodontia is the main culprit for the otherwise healthy population [1,12,13,14,15,16,17,18]. Osteoradionecrosis is a severe complication following radiotherapy for head and neck cancer patients. While osteoradionecrosis’s occurrence is on the decline, thanks to the use of Intensity-Modulated Radiation Therapy, it still lingers around 7% [19,20,21,22,23].

The diagnosis of pathological fractures of the mandible depends on both clinical examination and imaging. Pain, especially during mastication, difficulty in movement or structural deformation of the mandible, and changes to the patient’s occlusion may be indicative of a pathological fracture [24]. Pathological fractures of the mandible occur during mastication most of the time [12]. The initial imaging of choice for mandibular pathological fractures is an orthopantomogram (OPG) as it is a cheap and readily available examination. CT allows for more thorough structural visualization and possible surgical planning. Furthermore, it can contribute diagnostically to cases of suspected malignancy.

Treatment choice is determined by the etiology, location, and severity of the fracture. The body of the mandible is the most frequent fracture site, followed by the angle [12,17,24,25]. As no consensus or clear guidelines exist, there are several different approaches and subsequent treatment algorithms. The most crucial point is whether surgical intervention would be the most appropriate treatment choice or non-surgical management should be considered first [1,12,16,17,24,26,27,28,29,30,31].

Non-surgical management consists of pain management protocols (NSAIDS, opioids, corticosteroids), the administration of bisphosphonates and denosumab in metastatic bone disease, and radiotherapy for local control of malignant cases. Surgical management includes prophylactic fixation (for severely compromised structures or fractures awaiting biomechanical assessment), resection, and possible reconstruction with free flaps and bone grafts and/or internal fixation [32].

Osteoradionecrosis and pathological fractures related to malignant tumors pose a significant challenge in the management of head and neck oncology, primarily due to compromised bone quality, delayed healing, and the intricate anatomical factors involved [33]. Existing treatment protocols are hindered by several limitations, notably high rates of postoperative complications and inconsistent reconstruction outcomes, which impede effective patient recovery. Non-surgical approaches, including hyperbaric oxygen therapy or bisphosphonates, are often insufficient for managing advanced osteoradionecrosis or fractures related to tumors and are unable to prevent progression in severe mandibular ORN, necessitating surgical intervention [34]. Furthermore, the efficacy of hyperbaric oxygen in revitalizing necrotic bone is questionable, resulting in authors advocating for surgical management in advanced cases [35]. Current treatment protocols often lack individualization, failing to consider patient-specific factors such as radiation dose, tumor biology, or bone integrity. The variability in outcomes of ORN reconstructions due to the neglect of these factors calls for tailored treatment strategies [36]. The lack of consensus on the ideal timing for surgery following radiation or chemotherapy further complicates outcomes, as optimal conditions for bone and soft tissue recovery are frequently overlooked.

The development of evidence-based protocols for ORN prevention and management is hindered by the scarcity of high-quality clinical trials, with much of the current data originating from retrospective studies. This limitation hinders the establishment of robust treatment guidelines [37]. Additionally, the 2024 ISOO-MASCC-ASCO guidelines, which stress the importance of multidisciplinary approaches, fall short in providing detailed strategies for implementation, reflecting ongoing gaps in the evidence base [38].

Postoperative complications are largely associated with both systemic and local factors, particularly infections in hard and soft tissues or failures of the surgical hardware [39]. Patients with malignant tumors often present additional comorbidities, such as malnutrition or immunosuppression resulting from chemotherapy, which further exacerbate the risk of complications. These systemic factors are significant contributors to infections and delayed wound healing in ORN patients, emphasizing the critical need for thorough preoperative optimization [40].

An early diagnosis, along with proper planning and timely therapeutic intervention, is of paramount importance as it can greatly enhance patient treatment outcomes [41,42,43]. This study aims to present a series of 50 patients with pathological fractures of the mandible, their subsequent management, and the clinical and demographic data of this particular yet extremely diversified group of patients.

## 2. Materials and Methods

A retrospective, analytical study was conducted at the Oral and Maxillofacial Surgery Department of Aristotle University of Thessaloniki, Greece, a specialized center in the General Hospital G. Papanikolaou for the surgical treatment and rehabilitation of patients with head and neck cancer. The medical records and management of all patients with pathological fractures of the mandible treated in our clinic between January 2010 and December 2024 were examined.

The patients’ medical records were carefully examined by two of the researchers, who meticulously recorded the patient’s medical records for the following variables: age, gender, fracture location, length of hospital stay (after surgical intervention and total), type of management (surgical or conservative), type of surgical intervention (when applicable) etiology, pre- and post-surgery imaging, survival, alcohol and tobacco consumption history, and major comorbidities. The follow-up of these patients was recorded in an effort to establish the long-term outcomes of the intervention provided. Medical records with missing data were excluded from this study. Also, patients with a history of drug abuse were excluded from the present study. Lastly, patients presenting with a pathological fracture that had undergone therapeutic interventions elsewhere were excluded as well.

This retrospective study was performed in accordance with the tenets of the Declaration of Helsinki and the Medical Research Involving Human Subjects Act (WMO). Approval from the local Scientific Committee of the Hospital was obtained (Protocol No. 332/5-5-2025).

## 3. Results

### 3.1. Patient Characteristics

This study evaluated the medical records of 54 patients with pathological fractures of the mandible between January 2010 and December 2024. Four patients with a pathological fracture were excluded from the present study as they had significantly missing data.

The majority of the patients were male (78%, *n* = 39) and had an age > 60 years (58%, *n* = 29). The age range was 19–90 years. Tobacco (74%) and alcohol (60%) use was observed in most patients. Major comorbidities, such as cardiovascular disease, diabetes mellitus, history of malignancy, renal failure, COPD, etc., were also prevalent in this patient group (78%) (Table 1).

### 3.2. Fracture Characteristics

**Figure 1:**
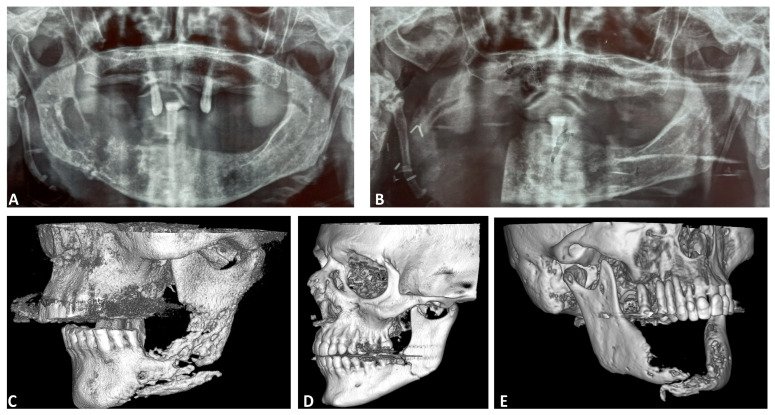
Imaging modalities of selected patients. (**A**) OPG of a female patient (78 years old) diagnosed with a mandibular pathological fracture of the right mandibular body due to MRONJ. (**B**) OPG of the same patient postoperatively, after right segmental mandibulectomy. (**C**) Three-dimensional anasynthesis of a CT scan of a male patient (83 years old) with a mandibular pathological fracture of the left angle due to osteoradionecrosis. (**D**) Three-dimensional anasynthesis of a CT scan of a male patient (50 years old) with a mandibular pathological fracture of the left angle after surgical extraction of a low-level-impacted third molar. (**E**) Three-dimensional anasynthesis of a CT scan of a female patient (64 years old) with an extended mandibular pathological fracture of the mandible due to malignancy.

The most frequent cause of mandibular pathological fracture was osteoradionecrosis (48%), followed by malignancy (22%), MRONJ (16%), and atrophy (14%) (Figure 1). Single fractures of the body of the mandible were the most frequent (56%) among all categories. Single fractures in general consisted of 68% of the cases. Multiple fracture sites were observed in 16 of the 50 cases, amounting to 20% unilateral and 12% bilateral fractures. Lastly, angle fractures represented 12% of the cases, while the ramus as a possible fracture location was only present in the multiple fracture group in six cases (12%). Fistulae were observed in 56% of the patients, with 18% of them having both intra- and extraoral fistulae. Regarding initial diagnostic imaging, OPG was used in 37 cases, CT in 25, and both were used in 14 cases. Biopsy was not performed in 30% of the fractures, while malignancy occurred 36% of the time (Table 2).

### 3.3. Intervention Characteristics

In total, 41 patients underwent surgery (82%), of whom 25 underwent mandibular resection, 14 mandibular resection and immediate reconstruction of the mandible, and 2 MMF. Hyperbaric oxygen therapy (10%) and the administration of Pentoxifylline (4%) were adjunct therapies in patients with osteoradionecrosis. As for post-treatment imaging, OPG was used in most cases (72%), with CT scan use observed in 14 cases, while both exams were performed for 12% of the patients. The average postoperative hospital stay was 11.5 days (minimum stay = 2 days, maximum stay = 49 days) (Table 3).

### 3.4. Outcomes

Mean patient survival after treatment was 27.6 months, with the minimum value being 1 month and the highest being 106 months. Complications such as fixation luxation, the loss of flap tissue, the contamination of osteosynthesis materials, etc., were observed in 54% of the patients (Table 4).

## 4. Discussion

Pathological fractures of the mandible, although rare, can significantly worsen a patient’s quality of life, having a negative impact on both psychological and physical welfare [2,44]. In the present study, most patients presenting with a pathological fracture were cancer patients, particularly head and neck cancer patients, who had undergone radiation therapy and subsequently developed osteoradionecrosis of the mandible. These findings are in line with the literature [1,12,13,16,17,25]. Recently, the occurrence of pathological fractures of the mandible associated with osteoradionecrosis has become increasingly frequent due to the wide use of adjuvant radiation therapy in head and neck cancer patients, in conjunction with better survival nowadays. Osteoradionecrosis of the jaws is considerably more common in continued tobacco users, in patients with diabetes mellitus, at the primary tumor site, with bony invasion of the primary tumor, and consequently in patients with pathological fractures. Ohori et al. (2023) [13] have determined the incidence of pathological fractures in patients with osteoradionecrosis to be 25.7%. As optimal treatment planning for osteoradionecrosis patients with mandibular pathological fractures is not standardized, surgical resection and free vascularized bone transfer have been proposed as the treatment of choice [26,27,28,30,31,41]. However, reconstructive surgery in patients with advanced-stage osteoradionecrosis is quite an endeavor since the quality of soft tissue healing is compromised due to both decreased blood supply and the loss of healthy tissues due to fistulae, occurring in more than 50% of this study’s patients. The devastating effects of osteoradionecrosis in the mandible have been speculated to be affected by the devascularization of it during selective neck dissection, where part of the facial artery is typically ligated. In addition, age-related vascular compromise of the mandible further contributes to the effects of radiotherapy, leading to complications such as pathological fractures [45]. Also, the depleted neck due to the absence of cervical vessels may hinder microvascular reconstruction [46,47]. Without doubt, appropriate treatment planning should consider the patient’s general health and nutritional status, as well as ruling out the possibility of recurrent cancer [25]. Cancer patients with osseous metastases who develop MRONJ have an increased survival rate, resulting in more patients developing pathological fractures due to MRONJ [48,49,50,51]. On the other hand, the incidence of osteomyelitis as a cause is not as frequent nowadays, probably due to the widespread use of and access to antibiotics. Fourteen percent of the pathological fractures had an underlying cause of atrophy of the mandible, which is in line with the early extraction of teeth being commonplace during the years of economic crisis in Greece. Bony lesions (cysts or benign tumors) can potentially lead to a pathological fracture, as they alter the adjacent bony structure, thus weakening the mandible and predisposing it to fractures.

Fractures resulting from cystic lesions are a rare occurrence [16]. There was only one pathological fracture due to iatrogenic reasons found in this retrospective study, clearly lower than other study findings [1,12,13,16,17,24]. This may be attributed to the fact that the institution of the authors mainly treats head and neck cancer patients. Furthermore, pathological fractures of the mandible following exodontia may be treated in the private sector more frequently than pathological fractures from other etiologies as they generally require shorter hospital stays and not particularly complex surgical procedures [14,18].

Diagnostic imaging plays an essential role in identifying pathological fractures of the mandible, guiding both diagnosis and treatment strategies. Conventional radiography, though commonly used, may not provide sufficient visibility of the underlying bone quality, structural integrity, or pathology present [52]. Instead, advanced imaging modalities such as CT provide detailed assessments of fracture patterns, enabling effective treatment planning. CT imaging is especially valuable in visualizing complex fractures associated with underlying pathologies and planning for reconstructive surgery [52,53]. Furthermore, high-quality CT imaging examinations are essential for presurgical treatment planning with the aid of 3D CAD/CAM technologies for the manufacturing of personalized surgical guides and/or internal fixation plates [54]. Thus, the integration of advanced imaging technologies in surgical planning is crucial, as it allows for a better understanding of the osseous anatomy, vascular supply, and existing lesions.

OPG and/or other types of radiographs of the visceral cranium, depending on the fracture site and type of surgical intervention, are the postoperative imaging of choice. CT is mainly performed when complications arise, or in future follow-ups when oncologic relapses are suspected.

Biopsy of the lesions of the pathological fracture is crucial, especially in cases of suspected malignancy, depending on the patient’s anamnesis. In our study, 36% of the biopsies were positive for malignancy. This alters treatment and surgical planning significantly, as well as overall prognosis.

A fracture of the mandibular body is the most frequent in the present study, followed by the angle of the mandible. Fractures involving solely the mandibular ramus were not observed at all and were only found in the multiple fracture group. These findings are in line with the demographic data of this study as well as the literature [4,24]. Most patients are male (78%) in their 6–7th decade of life. Tobacco and alcohol use and abuse are more prominent in groups of males, while they also exhibit poorer oral hygiene, thus resulting in an even more unfavorable set of conditions for the already compromised state of oral hygiene of head and neck cancer patients who have undergone radiation therapy [4,5].

As aforementioned, more than half the patients presented with extraoral or intraoral fistulas, which are a hindering factor in the surgical management of this group of patients. Fistula formation compromises the surrounding soft tissue healing capacity and complicates the surgical planning with a subpar postoperative healing process. In fistula cases where bone reconstruction is considered, soft tissue coverage and/or regional flaps should be considered. Free tissue transfer remains the gold standard for soft and hard tissue reconstruction, but as expected, patients with pathological fractures may be frail and not fit for this surgical intervention. In patients unfit for surgery who are managed conservatively, fistulas further worsen the quality of life.

The treatment of pathological fractures of the mandible should be tailored to the individual, considering the fracture’s causal factors, the fracture site, and the patient’s overall health status. Therapeutic options range from conservative management, including medications for pain relief and infection control, to various surgical procedures.

Surgical interventions remain the cornerstone in treating pathological fractures of the mandible. Surgical treatment was performed in 82% of the patients in the present study. Antimicrobials were administered to all the patients perioperatively, along with low-molecular-weight heparin as per standard practice. The use of innovative techniques such as virtual surgical planning can significantly enhance outcomes, particularly in complex cases involving extensive bone loss, with the employment of free fibula flaps for reconstruction following the resection of pathologic lesions showing reliable results in restoring functionality and facial aesthetics [55]. When electing for a surgical procedure as the first treatment choice for a pathological fracture, many factors must be considered first. These include, but are not limited to, patient-specific prognosis, health status, major comorbidities, recurrence of malignancy, concurrent treatments, nutritional status, tobacco and alcohol use/abuse, gender, and patient compliance [4,49,50,56,57]. Most of the 50 patients of this study (*n* = 39) had major comorbidities such as cardiovascular disease, diabetes mellitus, history of malignancy, renal failure, COPD, etc. These conditions further inhibit the patient’s healing mechanisms and potential for an uneventful postoperative period. More often than not, conservative management was selected as the first-choice treatment due to the compromised fitness for surgery. Furthermore, surgical planning may be altered by electing for a more minimal treatment approach, not involving the reconstruction of the mandible, avoiding a free tissue transfer procedure.

Adjunct therapies used were hyperbaric oxygen therapy and administration of Pentoxifylline in patients diagnosed with osteoradionecrosis. While the current consensus for hyperbaric oxygen therapy is that it should not be used routinely for the treatment of osteoradionecrosis, it could be beneficial for a subgroup of patients where other treatment options have been fruitless [58,59,60,61]. On the other hand, the use of Pentoxifylline could potentially be more promising, although its use and dosage need to be standardized [62,63,64].

Half the patients presented complications in future follow-ups. This can be attributed to the compromised healing process, especially in cancer patients with major comorbidities. The past history of radiation therapy also hinders the healing process in the postoperative period. Patients should be followed closely to ensure that possible complications are diagnosed early, allowing for immediate intervention and treatment.

The median overall survival after therapeutic intervention (surgical or not) was 27.6 months, which can be attributed to the compromised health status of most individuals presenting with a pathological fracture in our study. As aforementioned, the majority of the patients treated were cancer patients, either head and neck cancer patients with osteoradionecrosis/primary malignancy/malignancy recurrence or patients with MRONJ for other types of malignancies. Also, 78% of the patients had major comorbidities too. In the case series reviewed, there was no mention of overall survival after intervention or diagnosis.

The potential limitations of the present study can be attributed to the inherent limitations of retrospective studies. Additionally, subgroup analysis could not be performed due to the heterogeneity of the data and the small number of patients in each subgroup. Furthermore, as mentioned in the etiology segment, there could be an underreporting of pathological fractures, especially those following exodontia, from the private sector. Also, as our department mainly treats head and neck cancer patients, center-specific bias cannot be excluded.

Lastly, there is a glaring gap in the present literature for a widely accepted classification system and a clear consensus for a cohesive algorithmic management for mandibular pathological fractures as opposed to other possible pathological fracture sites [10]. This issue must be addressed in order to enable more universally accepted treatment planning that leads to elevated therapeutic results for the patients treated.

## 5. Conclusions

This longitudinal retrospective analysis of patients presenting with pathological fractures of the mandible delineates the considerable clinical intricacies and therapeutic challenges inherent to this rare condition, predominantly encountered among individuals with head and neck cancer complicated by osteoradionecrosis. The data elucidate the extensive impact of such fractures on functional capacity, facial aesthetics, and psychological well-being, further aggravated by comorbidities and complications, including fistulae formation and impaired wound healing. Surgical intervention remains the principal therapeutic approach, individualized according to patient-specific factors. Technological advances, such as high-resolution imaging and virtual surgical planning, have notably contributed to improved outcomes. Nevertheless, the absence of standardized treatment protocols and a universally recognized classification system for mandibular pathological fractures constitutes a significant deficiency in the existing literature. There is a pressing need for future research to establish comprehensive management algorithms and undertake prospective, multicenter investigations aimed at optimizing therapeutic strategies and enhancing patient quality of life.

## Figures and Tables

**Table 1:** Patient characteristics.

Patient Characteristics	
Variable	Number of Patients (%)
Age	
<60 years	21 (42%)
>60 years	29 (58%)
Median Age	66.4 years
Sex	
Male	39 (78%)
Female	11 (22%)
Major comorbidities	
Yes	39 (78%)
No	11 (22%)
Smokers	
Yes	37 (74%)
No	13 (26%)
Alcohol use	
Yes	30 (60%)
No	20 (40%)

**Table 2:** Fracture characteristics.

Fracture Characteristics	
Variable	Number of Patients (%)
Etiology	
Medication-related osteonecrosis of the jaws	8 (16%)
Osteoradionecrosis	24 (48%)
Primary malignancy	11 (22%)
Recurrence of malignancy	7 (14%)
Benign tumours	2 (4%)
Cysts	1 (2%)
Osteoporosis	1 (2%)
Mandibular atrophy	7 (14%)
Osteomyelitis	4 (8%)
Iatrogenic (after tooth extractions)	1 (2%)
Fracture location	
Body only	28 (56%)
Angle only	6 (12%)
Ramus (as part of multiple locations)	6 (12%)
Multiple (unilateral)	10 (20%)
Multiple (bilateral)	6 (12%)
Presence of fistula	
No	22 (44%)
Yes, intraoral	9 (18%)
Yes, extraoral	10 (20%)
Yes, both	9 (18%)
Imaging	
OPG	37 (74%)
CT	25 (50%)
Both	14 (28%)
Biopsy	
No	15 (30%)
Yes, inflammation	17 (34%)
Yes, malignancy	18 (36%)

**Table 3:** Intervention characteristics.

Intervention Characteristics	
Variable	Number of Patients (%)
Surgical intervention	
Yes	41 (82%)
No	9 (18%)
Type of surgical intervention	
Mandibular resection	25 (61%)
Mandibular resection and reconstruction	14 (34%)
MMF	2 (5%)
Adjunct Therapies	
Hyperbaric Oxygen Therapy	5 (10%)
Pentoxifylline	2 (4%)
Length of hospital stay (days)	2–49 [median 11.5]
Postoperation imaging	
OPG	36 (88%)
CT	14 (34%)
Both	6 (15%)

**Table 4:** Outcomes.

Outcomes	
Variable	Number of Patients (%)
Complications	
Yes	27 (54%)
No	23 (46%)
Overall survival (months)	1–105 [median 27.6]

## Data Availability

Data are available upon request.

## Abbreviations

The following abbreviations are used in this manuscript:

MRONJ	Medication-Related Osteonecrosis of the Jaws
OPG	Orthopantomogram
3D	Three-Dimensional
CT	Computed Tomography
NSAIDs	Nonsteroidal Anti-Inflammatory Drugs
MMF	Maxillo-Mandibular Fixation
CAD/CAM	Computer-Aided Design/Computer-Aided Manufacturing
COPD	Chronic Obstructive Pulmonary Disease
